# Proteomic Evidence of Biological Aging in a Child with a Compound Heterozygous *ZMPSTE24* Mutation

**DOI:** 10.1002/prca.201800135

**Published:** 2018-12-27

**Authors:** Angela K Lucas‐Herald, Petra Zürbig, Avril Mason, Esther Kinning, Catriona E Brown, Bahareh Mansoorian, William Mullen, Syed Faisal Ahmed, Christian Delles

**Affiliations:** ^1^ Institute of Cardiovascular and Medical Sciences University of Glasgow Glasgow G12 8TA UK; ^2^ Developmental Endocrinology Research Group School of Medicine University of Glasgow Glasgow G51 4TF UK; ^3^ Mosaiques Diagnostics GmbH Rotenburger Str. 20 30659 Hannover Germany; ^4^ Department of Clinical Genetics Queen Elizabeth University Hospital Glasgow G51 4TF UK

**Keywords:** biomarkers, capillary electrophoresis‐mass spectroscopy, progeria

## Abstract

**Background:**

Progeria‐like syndromes offer a unique insight into aging. Here the case of a boy affected with mandibuloacral dysplasia and compound heterozygous mutations in *ZMPSTE24* is presented.

**Methods:**

Capillary electrophoresis‐mass spectroscopy is used for proteome analysis to analyze peptides previously found to be differentially regulated in chronic kidney disease (273 peptides defining the CKD273 classifier), coronary artery disease (238 peptides defining the CAD238 classifier), and aging (116 peptides defining the AGE116 classifier).

**Results:**

No evidence of renal disease is identified. Although the boy has no overt cardiovascular disease other than a raised carotid intima media thickness relative to his age, a proteomic classifier for the diagnosis of coronary artery disease is mildly raised. The biological age based on the proteomic AGE116 classifier is 24 years compared to the chronological ages of 5 and 10 years. In contrast, a control group of healthy children has a significantly lower (*p* < 0.0001) calculated mean age of 13.

**Conclusion:**

Urinary proteomic analysis is effective in confirming advanced biological age and to identify early evidence of renal or cardiovascular damage. This case highlights the value of proteomic approaches in aging research and may represent a method for non‐invasive monitoring of the effects of early aging.

Progeria‐like syndromes are rare disorders resulting in premature aging. With an estimated prevalence of 1 in 20 million people, the most common of these syndromes is Hutchinson–Gilford progeria syndrome (HGPS) (OMIM 176670), caused by a de novo mutation in the *LMNA* gene.^1^ Patients with a similar phenotype have reportedly suffered loss of function mutations in *ZMPSTE24* (zinc metalloproteinase STE24).^2^ The associated phenotype is attributed to accumulation of prelamin A, as ZMPESTE24 is a membrane protein which is critical in the posttranslational processing of prelamin A to mature lamin A.^3^ Affected patients had characteristic facial features (micrognathia, sparse thin hair, small pinched nose, enlarged fontanelles and delayed dentition) and postnatal growth delay followed by the development of lipodystrophy and renal and vascular complications.^4^ Animal models of these mutations demonstrate some of the key hallmarks of aging including altered chromatin organization, defective extracellular matrix production, and cellular senescence.^5^ The interaction between genetic and environmental factors in progeria‐like syndromes can modify disease processes, leading to different phenotypes and disease progression despite similar genetic predisposition. In this context, analysis of protein biomarkers can provide a better description of the disease process at a given point in time compared to analysis of genetic factors that remain largely stable throughout life.

Urinary proteomics offers the potential to screen large numbers of urinary biomarkers, which can then be used for non‐invasive early detection of disease.^6^ Repeated blood sampling in children can be challenging and distressing for children.^7^ Although there has been interest in whether it would be possible to identify potential urinary biomarkers regarding the progression of disease in progeria‐like syndromes, to date none have been successfully identified. Prelamin A is located in the nuclear lamina, making it challenging to identify in the urine. In 2006, Adachi et al.^8^ were able to identify only one fragment of Lamin A/C in one sample with one specific method. As such, alternative urinary biomarkers are still being sought.

In 2009, the low‐molecular‐weight urinary proteome of 324 healthy individuals between the ages of 2–73 years was analyzed, identifying age related changes in the secretion of 325 urinary peptides, the majority of which were associated with renal development before or during puberty and 49 peptides, which were related to aging. Even in apparently healthy individuals, there was evidence of markers of chronic age‐related kidney disease, which may be pre‐clinical. Further analysis demonstrated that 13/49 peptides were most robustly associated with aging.^9^

More recently, urinary proteomes of 1227 healthy and 10 333 diseased individuals between 20–86 years of age were investigated.^10^ A total of 116 peptide biomarkers were identified that significantly correlated with age in the healthy cohort. These peptides predominantly originated from collagen, uromodulin, and fibrinogen. While most fibrillar and basement membrane collagen fragments showed a decreased age‐related excretion, uromodulin, beta‐2‐microglobulin, and fibrinogen fragments showed an increased urinary excretion. Using the same capillary electrophoresis‐mass spectroscopy (CE‐MS) data it is also possible to focus on peptides that are involved in specific disease processes such as coronary artery disease and chronic kidney disease.^11^, ^12^

It is clear that urinary proteomics may provide useful insights into vascular aging. Here we present the urinary proteomic profile of a boy with a rare autosomal recessive progeria‐like syndrome caused by a *ZMPSTE24* mutation.^13^ He was born at term with a birthweight of 3.71 kg (50th centile). There was no significant family history in his parents or his two older siblings. He required pediatric review in his first months because of failure to thrive (0.4th centile). At 10 months, he was noted to have abnormal skull development. Bone biochemistry was normal but a skull X‐ray demonstrated widely patent fontanelles and wormian bones over the occipital area. On further review at the age of 1.8 years, he had mid facial hypoplasia with sparse thin hair, thin skin, prominent vasculature, normal clavicles, no plantar arch, inward bowing of his legs, short stature (2nd centile, below mid parental range) and delayed dental eruption. Skeletal survey confirmed osteopenic bones. Due to clinical suspicion of mandibuloacral dysplasia, he had genetic testing which identified one nonsense and one missense mutation in *ZMPSTE24*, c.202C>T/c.743C>T predicting p.R68X/P248L.

The child is currently aged 11.9 years. He is growing along the 2nd centile and on no regular medications, other than multivitamin drops containing 400 units ergocalciferol. He has osteonecrosis of his left hip that has not required any surgical intervention. His bone density is low (total body density −2.2; lumbar spine −1.3). His renal function and echocardiogram are normal and his blood pressure has remained on the 50th centile for height and age. Carotid intima media thickness (CIMT) measurements of the common carotid artery at the age of 10.7 years, taken as previously described^14^ using the Siemens Acuson Sequoia 512 (California, USA) demonstrated that his CIMT was 0.47mm, which is on the 95th centile for age.^15^ Pulse wave velocity measured using the SphygmoCor system (AtCor Medical Ltd, Sydney, Australia) was on the 25th centile for height and age.^16^

Spot urine samples of the case were collected at two time points in 2012 (age 5 years) and 2017 (age 10 years). Furthermore, spot urine samples of two healthy controls at the age of 5 years and two healthy controls at the age of 10 years were used. The preparation of the urine samples, CE‐MS analysis, and data processing were performed as described previously in detail^12^ and although the samples were from different time points, no degradation would be expected as demonstrated previously by Zürbig et al.^17^ Mass spectral ion signals representing identical molecules at different charge states were deconvoluted into single masses using the MosaiquesVisu software.^18^ To achieve high mass accuracy, deconvoluted mass signals were calibrated based on a Fourier‐transform ion cyclotron resonance mass spectrometer (Bruker Daltonics Apex Qe instrument equipped with a 12‐tesla magnet and an Apollo II ion source) with accurate masses (mass deviation of 1 ppm) as described.^12^, ^19^ In parallel, CE‐migration time was normalized by local regression and signal intensities using internal standards.^20^ All analyses passed quality control. Classification of the urine samples based on a comparison of the levels of biomarkers detected in the urine of the individual patient to the levels of those biomarkers identified using SVM‐based classifier, the MosaDiagnostics software (version 1.4.0), and the previously defined classifiers for chronic kidney disease and coronary artery disease were used.^21^ Furthermore, with the previously identified 116 aging peptide biomarkers,^10^ an SVM‐based classifier (AGE116) was generated based on randomly selected 45 young (age: 20–29 years) and 45 old (age: >60 years) healthy individuals of the original cohort. The correlation between the classification scores of this classifier and the age of the subjects resulted in a coefficient of rho = 0.77 (95% CI: 0.67–0.84; *p* < 0.0001). The classifier was further validated with the rest of the original cohort and resulted in a correlation of rho = 0.64 (*p* < 0.0001). A *p*‐value of 0.1 was considered statistically significant due to the low number of samples.

Individual peptide data from the CE‐MS runs are available in Table S1, Supporting Information. We focused on the analysis of peptides that have been previously found in other studies to be differentially regulated in coronary artery disease (238 peptides defining the CAD238 classifier)^22^ and chronic kidney disease (273 peptides defining the CKD273 classifier)^12^ and were found to be associated with aging (116 peptides defining the AGE116 classifier). Classifier scores are presented in Table 1. The CKD273 classifier was within the normal range at both time points, providing no evidence of subclinical renal disease in this patient. The CAD238 classifier for coronary artery disease was above the threshold of −0.140 in 2012 and in the normal range but close to the threshold in 2017. We also constructed a regression model for the AGE116 classifier based on previously published data^10^ so that we were able to transform the score into an estimated biological age. In 2012, at the chronological age of 5 years, the biological age based on AGE116 classifier was 23.8 years, and it remained 24.1 years in 2017 when the chronological age was 10 years.

**Table 1:** Proteomic classifier scores. Cut‐off values for CAD238 and CKD237 for diagnosis of coronary artery disease and chronic kidney disease are −0.140 and 0.343, respectively, with higher values representing presence and lower values absence of disease

	2012	2012	**2012**	2017	2017	**2017**	Age 5	Age 5	**Age 5**	Age 10	Age 10	**Age 10**
	Sample 1	Sample 2	**Mean**	Sample 1	Sample 2	**Mean**	Control 1	Control 2	**Mean**	Control 3	Control 4	**Mean**
**CAD238**	0.283	−0.198	**0.043**	−0.018	−0.161	−**0.090**	−0.270	−0.225	−**0.248**	−0.537	−0.289	−**0.413**
**CKD273**	−0.412	−0.363	−**0.388**	−0.481	−0.319	−**0.400**	−1.276	−0.442	−**0.859**	−0.849	−0.871	−**0.860**
**AGE116**	−1.104	−0.960	−**1.032**	−0.988	−1.047	−**1.018**	−1.634	−1.541	−**1.588**	−1.527	−1.589	−**1.558**
**Predicted age**	22.4	25.3	**23.8**	24.7	23.5	**24.1**	11.6	13.5	**12.5**	13.8	12.5	**13.1**

To compare the estimated biological age which was calculated with the AGE116 classifier, we used a control group of two healthy children at the age of 5 and two at the age of 10, extracted from the Human Urinary Database.^21^ The estimated biological age of the younger control group was 12.5 years and for the older control group was 13.1 years which was (at both ages) significantly (*p* < 0.0001) lower than the estimated biological age of the case subject (see Table 1)

Furthermore, we compared expression of individual peptides that define the AGE116 classifier of the case samples with the mean expression of these peptides in the above control group (Figure 1). We found nine peptides which were significantly different in the mean expression of the case samples compared to controls (Table S2, Supporting Information). Six of these showed a higher expression in the urine of healthy controls than in the case samples; five derived from collagen alpha‐1 type I and III. A decrease in the mean expression of collagen peptides may be the result of the accumulation of ECM during aging secondary to reduced ECM degradation.^10^ Although this number of collagen‐derived peptides may appear low, in the study by Nkuipou‐Kenfack et al., only 55 collagen derived peptides were identified from a sample of 1227 individuals,^10^ suggesting that our output is reasonable given the relatively smaller number of samples. Furthermore, we identified three peptides, which are fragments of fibrinogen alpha and beta chain and of uromodulin with a higher mean expression in the control than in case samples. The urinary excretion of these peptides showed a positive correlation with age.^23^

**Figure 1:**
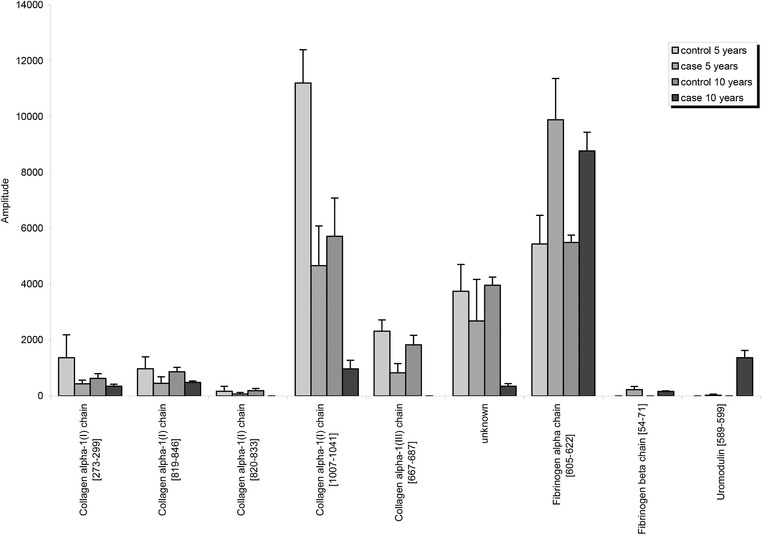
Mean amplitude of significant peptides.

Although these proteomic markers are not currently validated as routine clinical parameters, there is accumulating evidence of their diagnostic potential.^24^ Given that our data demonstrate that urinary peptide analysis by CE‐MS does correlate effectively with clinical phenotype, this could be a non‐invasive alternative to plasma protein analysis. The proteomic age classifier we have generated demonstrates a clear discrepancy between estimated biological age and chronological age. However, the estimated biological age identified as 24 years may be less than expected in light of the boy's aged musculoskeletal phenotype. This age classifier provides an estimate of overall age and is not organ specific. More advanced musculoskeletal age can therefore be outweighed by other clinical parameters such as normal renal function with no discrepancy between chronological and estimated biological age.

One potential limitation of this work is that the classifiers used have been calculated using adult data. The urinary proteome changes significantly during puberty^9^; therefore, it is difficult to find peptides from a pediatric population which correlate with age. The classifiers we have used are therefore the most appropriate currently available to us. Our group has previously used the CKD273 classifier in children with chronic kidney disease and have found it to be accurate, with a different reference range to the adult population. Our group has not previously used markers associated with CAD in children.

To conclude, we have demonstrated that urinary proteomic analysis can be used to confirm advanced estimated biological age in a boy with a progeria‐like syndrome. Our data correlate with the patient's physical characteristics and although in our boy there are no immediate consequences of this finding, with future research, proteomics could represent a method for non‐invasive monitoring of similarly affected patients for clinical deterioration. The progeria‐like syndromes offer a unique insight into normal and pathological aging processes that affect multiple organ systems at the same time that have individually been subject to previous and ongoing proteomic studies. Our case, therefore, highlights the value of proteomic approaches in aging research and provides insights into molecular features of multimorbidity.

## Conflict of Interest

P.Z. is employed by Mosaiques Diagnostics GmbH, Hannover, Germany; the company develops proteomic biomarkers for prediction and diagnosis of human diseases. The other authors have nothing to disclose.

## Supporting information

Supporting Information
